# Jurisdiction in Employment Matters Post-Brexit

**DOI:** 10.1093/ojls/gqag017

**Published:** 2026-05-20

**Authors:** Uglješa Grušić

**Keywords:** jurisdiction, employment, Brexit, Civil Jurisdiction and Judgments Act 1982, Employment Tribunal Procedure Rules 2024, private international law

## Abstract

This article deals with the jurisdiction of the civil courts and employment tribunals in employment matters post-Brexit. It focuses on the somewhat defective transposition of the rules of jurisdiction in employment matters of the Brussels Ia Regulation in sections 15A and 15C–15E of the Civil Jurisdiction and Judgments Act 1982 and the unclear relationship between these rules and other rules that regulate jurisdiction in employment matters, most importantly employment tribunal procedure rules. By deconstructing the current law, this article discloses its considerable complexity and discusses possibilities for improvement.

## 1. Introduction

A prominent feature of the private international law of employment in the UK during the country’s membership of the EU was the operation of the European regime of protection of employees as weaker parties, including by special jurisdiction rules, currently set out in the Brussels Ia Regulation^1^ and the 2007 Lugano Convention.^2^

The UK government found that the Brussels Ia Regulation’s regime of protection of employees was superior to the traditional UK law and it decided to transpose it after the country’s withdrawal from the EU by means of sections 15A and 15C–15E of the Civil Jurisdiction and Judgments Act 1982^3^ (CJJA 1982 rules). The goal of these provisions is to ‘retain to a large degree the … employee-friendly approach of the Brussels regime’.^4^

As often, however, the devil is in the detail. Before Brexit, the law of jurisdiction in employment matters was formed of several clearly separated tracks: (i) the European regime, comprised of the jurisdiction rules of (a) the Brussels Ia Regulation and (b) the 2007 Lugano Convention; and (ii) the traditional English and Welsh, Scottish and Northern Irish laws, which determined the jurisdiction of courts where the jurisdiction rules of the Brussels Ia Regulation and the 2007 Lugano Convention did not apply. In England,^5^ the traditional law was comprised of: (i) rules on service in the jurisdiction; (ii) rules on service outside the jurisdiction; and (iii) rules determining the jurisdiction of employment tribunals. The current law is also formed of several tracks (in England: (i) the CJJA 1982 rules; (ii) rules on service in the jurisdiction; (iii) rules on service outside the jurisdiction; and (iv) rules determining the jurisdiction of employment tribunals), but the relationship between them is unclear.

The lack of clarity is symptomatic of deeper problems with the transposition of the Brussels Ia Regulation’s rules. The UK government has failed to appreciate some of the nuances of these rules and of the ‘hidden depths’^6^ of the traditional law, onto which the CJJA 1982 rules have been superimposed. The lack of clarity and the deeper problems with the transposition of the Brussels Ia Regulation’s rules, in turn, undermine the goal of the CJJA 1982 rules. This matters because access to remedy and the enforcement of employment rights in cases with a foreign element depend on the clear and coherent operation of jurisdiction rules.

To make this argument, this article deconstructs the current law of jurisdiction in employment matters by addressing a series of questions in sections 3–8. Do the CJJA 1982 rules apply before employment tribunals (section 3)? If so, do they exclusively and exhaustively determine the international jurisdiction of employment tribunals (section 4)? Which rules allocate jurisdiction to hear employment cases within the UK (section 5)? How should courts deal with the defective transposition of the Brussels Ia Regulation’s rules (section 6)? Can the doctrine of *forum non conveniens* apply (section 7)? What are the effects of jurisdiction agreements (section 8)? The picture that emerges from this discussion is one of considerable complexity, which is not fully reflected in either the existing accounts of the English private international law of employment or case law. The last section (section 9) discusses possibilities for improvement.

Some background information needs to be presented first, that is: the different judicial forums that can hear employment cases in England; the different types of claims that can be brought in England; the different meanings of the term ‘jurisdiction’ in the English private international law of employment; and a brief overview of the law of jurisdiction in employment matters before and after Brexit (section 2).

## 2. Background

### A. Civil Courts and Employment Tribunals

In England, employment cases can be heard by the civil courts, that is, the High Court and the county court, and employment tribunals.

Unlike the High Court, employment tribunals have no inherent jurisdiction. As creatures of statute, their powers are derived from and limited to what statute has conferred. Ultimately, their jurisdiction is derived from the Employment Tribunals Act 1996 (ETA 1996), which provides in section 2 that ‘Employment tribunals shall exercise the jurisdiction conferred on them by or by virtue of this Act or any other Act, whether passed before or after this Act’. Rights given to employees by or by virtue of employment statutes forming part of English law (statutory employment rights) are enforceable before employment tribunals.^7^ The Employment Tribunals Extension of Jurisdiction (England and Wales) Order 1994^8^ (1994 Order) extends the jurisdiction of employment tribunals to certain claims for breach of contract. Employment tribunals hear cases brought by employees, with the limited exception of contractual counterclaims, which can be brought by employers.^9^

The civil courts can hear cases for breach of contract and common law rights brought by either employees or employers.

Collectively, the civil courts and employment tribunals are referred to as ‘courts’ in this article.

### B. Common Law and Statutory Claims

There are, therefore, two basic types of claims that can be brought in England. Claims for breach of contract and common law rights (common law claims) can be brought in the civil courts and, within the confines of the 1994 Order, employment tribunals. Claims for breach of statutory employment rights (statutory claims) can only be brought in employment tribunals.

### C. Meanings of ‘Jurisdiction’

As is clear from the preceding text, the term ‘jurisdiction’ has different meanings in employment cases.

In an influential article published in 2010,^10^ Professor Merrett explained how it is very common in employment cases to hear ‘jurisdiction’ being used in what are, in fact, three different contexts:

1. in cases with a foreign element, the question of private international law whether England rather than any other country is the appropriate jurisdiction in which the case should be heard, referred to as ‘international jurisdiction’;
2. in domestic cases, or in cases with a foreign element where England has international jurisdiction, the question as to which domestic court should hear the case, referred to as ‘domestic jurisdiction’; and
3. the question of substantive scope of statutory employment rights, most importantly the question of their express or implied territorial limits, referred to as ‘territorial scope’. Courts often refer to this question as ‘territorial jurisdiction’.^11^

The separation of these three questions has received judicial endorsement, starting with Langstaff P in *Simpson v Intralinks Ltd*.^12^

However, these meanings of the term ‘jurisdiction’ are somewhat ambiguous and should be defined and distinguished more clearly.

First, there is the question of adjudicatory jurisdiction with respect to the parties to the dispute, often referred to as ‘personal jurisdiction’. This question can be further divided into questions of ‘international’ and ‘intra-UK jurisdiction’. ‘International jurisdiction’ concerns the power of a UK court to hear a case that has a connection with a country outside the UK. ‘Intra-UK jurisdiction’ concerns the power of a UK court to hear a case that has connections with more than one part of the UK.

Second, there is the question of adjudicatory jurisdiction with respect to the subject matter of the dispute, often referred to as ‘subject-matter jurisdiction’. This question is particularly important in employment cases because employment tribunals are only empowered to hear statutory employment claims and some common law claims, and the civil courts cannot hear statutory employment claims. Courts refer to this meaning of the term ‘jurisdiction’ as ‘cause of action jurisdiction’.^13^

Third, the question of ‘territorial scope’ is not a question of adjudicatory jurisdiction, but one of the substantive scope of UK employment law. It is for this reason that this article avoids using the term ‘territorial jurisdiction’.

The different meanings of the term ‘jurisdiction’ are closely related. The questions of ‘international’ and ‘intra-UK jurisdiction’ can both arise in a case that has connections with more than one part of the UK and a country outside the UK, and, as discussed in section 5 below, the CJJA 1982 rules regulate both ‘international’ and ‘intra-UK jurisdiction’. The question of ‘territorial scope’ flows into the question of ‘cause of action jurisdiction’, as a result of section 2 ETA 1996 read together with legislation providing that statutory employment rights are enforceable before employment tribunals. Since employment tribunals are the natural forum for statutory claims, there are arguments that *forum non conveniens* should not be used to deprive employment tribunals of their ‘international jurisdiction’^14^ and that the ‘principle’ *ubi jus, ibi remedium* (where there is a right, there should be a remedy) should result in the ‘international jurisdiction’ of employment tribunals whenever the case is within the ‘territorial scope’ of a statutory employment right.^15^

Given the centrality of the ‘principle’ *ubi jus, ibi remedium* to the reasoning of the recent judgments of the Employment Appeal Tribunal in *Bhatti*^16^ and *Lapinski*,^17^ a few words should be said about it before proceeding further.

In *Bhatti*, Kerr J accepted that ‘domestic statutes which have territorial application to a claim do themselves confer international jurisdiction’, unless qualified or displaced by primary legislation.^18^ Two authorities were cited in support of this assertion. First, *Pervez v Macquarie Bank Ltd*, where the Employment Appeal Tribunal stated that ‘it is … wrong in principle that a group of employees, however limited, should notionally enjoy protections which they cannot in fact enforce’.^19^ Second, the following *dictum* of Lord Hoffmann in *Lawson*:

Putting the question in the traditional terms of the conflict of laws, what connection between Great Britain and the employment relationship is required to make section 94(1) [of the Employment Rights Act 1996 (ERA 1996), giving the right not to be unfairly dismissed] the appropriate choice of law in deciding whether and in what circumstances an employee can complain that his dismissal was unfair? The answer to this question will also determine the question of jurisdiction, since the Employment Tribunal will have jurisdiction to decide upon the unfairness of the dismissal if (but only if) section 94(1) is the appropriate choice of law.^20^

In *Lapinski*, Auerbach J agreed with Kerr J.^21^

However, Kerr J’s analysis is not supported by the cited authorities and is based on a category error. In *Pervez*, the Employment Appeal Tribunal dealt with the interpretation of regulation 19 of the Employment Tribunals (Constitution and Rules of Procedure) Regulations 2004^22^ (ET(CRP)R 2004), which, as discussed in detail in section 4B below, at the time regulated the international jurisdiction of employment tribunals. Second, Lord Hoffmann’s *dictum* from *Lawson* should be read together with a subsequent passage from the same case, where his lordship noted that

no one supported the solution which had found favour with the Employment Appeal Tribunal in the *Serco* case, namely that section 94(1) now applies to employment relationships anywhere in the world, subject only to the Employment Tribunal having *personal jurisdiction over the defendant under the Employment Tribunals (Constitution and Rules of Procedure) Rules 2001* [the predecessor of ET(CRP)R 2004].^23^

The fact that his lordship did not state anywhere that the suggestion that personal jurisdiction depended on the application of the 2001 Rules (where the jurisdiction rules of the Brussels I Regulation and the Lugano Convention did not apply) suggests that he considered the questions of ‘international jurisdiction’ and ‘territorial scope’ to be separate. In other words, Lord Hoffmann’s *dictum* can be read as indicating that the question of ‘territorial scope’ flows into the question of ‘cause of action jurisdiction’ and is related to the choice-of-law/applicable law query, and, as such, is conceptually different from ‘international jurisdiction’, a species of personal jurisdiction. Further support that Kerr J’s analysis is based on a category error can be derived from the fact that if the ‘principle’ *ubi jus, ibi remedium* existed as a matter of principle in the English law of jurisdiction, then other statutory rights would have the same effect and Civil Procedure Rules Practice Direction 6B, paragraph 3.1(20)^24^ would be unnecessary.

### D. Pre-Brexit Law: An Outline

As mentioned in the introduction, the pre-Brexit law of jurisdiction in employment matters was formed of several clearly separated tracks.^25^

If the jurisdiction rules of the Brussels Ia Regulation applied (where the defendant was domiciled in an EU Member State, where the habitual place of work was in a Member State, where, if there was no habitual place of work, the engaging place of business was in a Member State or where there was non-consensual or consensual exclusive jurisdiction),^26^ they determined whether English courts had jurisdiction. Similarly, if the jurisdiction rules of the 2007 Lugano Convention applied (from the perspective of English courts, where the defendant was domiciled in Iceland, Norway or Switzerland, or where there was exclusive jurisdiction in one of these countries),^27^ they determined whether English courts had jurisdiction. Under the Brussels Ia Regulation^28^ and the 2007 Lugano Convention,^29^ employees can choose from a number of forums to sue their employers, including the courts of the employer’s domicile and the courts for the habitual place of work, whereas employers are essentially limited to suing their employees in the courts of the employee’s domicile. Party autonomy, that is, the ability of the parties to agree on the competent court, is restricted. Importantly, the jurisdiction rules of the Brussels Ia Regulation and the 2007 Lugano Convention applied to determine the jurisdiction of both the civil courts and employment tribunals.^30^

Where the jurisdiction rules of the Brussels Ia Regulation and the 2007 Lugano Convention did not apply, the distinction between common law claims and statutory claims was of fundamental importance. Since the jurisdiction of employment tribunals to hear certain contract claims is identical to, and concurrent with, that of the civil courts,^31^ the jurisdiction of courts to hear common law claims depended on rules on service in and outside the jurisdiction.^32^ There were separate rules determining the jurisdiction of employment tribunals to hear statutory claims, although there was some uncertainty as to what those rules were. The predominant view was that the relevant jurisdiction rules were found in employment tribunal procedure rules,^33^ that is, rule 8(2) and (3) of the Employment Tribunals Rules of Procedure, annexed as schedule 1 to the Employment Tribunals (Constitution and Rules of Procedure) Regulations 2013^34^ (ETRP 2013) at the time of Brexit. Another view was that the fulfilment of the territorial scope requirement conferred international jurisdiction and that employment tribunal procedure rules only dealt with intra-UK jurisdiction.^35^ Jurisdiction agreements were given effect as part of the *forum (non) conveniens* discretion.^36^

### E. Current Law: An Outline

As an instrument that allocates jurisdiction and regulates the mutual recognition and enforcement of judgments in civil and commercial matters among EU Member States on a reciprocal basis, the Brussels Ia Regulation was not retained as assimilated EU law after Brexit. Since the EU concluded the 2007 Lugano Convention on behalf of all Member States except Denmark, the convention ceased to be applicable to the UK after Brexit. The UK tried to keep the benefits of the European regime of jurisdiction and free movement of judgments in civil and commercial matters by applying to accede to the 2007 Lugano Convention in its own right. However, the European Commission has taken the view that accession is closed to non-EU states that are not part of the European Free Trade Association/European Economic Area.^37^

Having found that the Brussels Ia Regulation’s regime of protection of employees was superior to the traditional UK law, the UK government decided to transpose it by means of sections 15A and 15C–15E CJJA 1982. According to the Explanatory Memorandum to the amendments to the CJJA 1982 introducing these provisions:

The rationale for retaining and restating the approach to jurisdiction in … employment cases contained in the Brussels Ia Regulation … reflects the protective nature of these rules towards parties who are traditionally seen as economically weaker and perhaps less legally aware than their opponents (so … employers). The rules ensure that the … employee should in general not have to sue, or be sued, in a jurisdiction which is unfamiliar to him in terms of, for example, language. These rules applied not only to … employers, domiciled in the EU, but also those who were not so domiciled, and this approach is continued. These protective rules are not available in the common law and statutory provision of England and Wales and Northern Ireland, and only to a more limited extent in Scotland, apart from the Brussels regime, and so the Government has chosen to retain them.^38^

The CJJA 1982 rules apply only in relation to proceedings whose subject matter is within the scope of the Brussels Ia Regulation^39^ and relating to an individual contract of employment.^40^

On the one hand, there is one set of jurisdiction rules applicable when employees act as *claimants*. The employer may be sued by the employee in England: (i) if the employer is domiciled in England; *^41^ (ii) if the habitual place of work is or last was in England; *^42^ (iii) if there is no habitual place of work, if the engaging place of business is or was in England; *^43^ (iv) on a counterclaim, if the original claim against the employee domiciled in England is pending in England;^44^ (v) if the employer is domiciled in another part of the UK, as regards a dispute arising out of the operations of the employer’s branch, agency or other establishment, if the establishment is in England;^45^ (vi) if the employer is domiciled in another part of the UK and a co-defendant is domiciled in England, provided the claims against the co-defendants are sufficiently closely connected;^46^ (vii) if the employer is domiciled outside the UK, under any other rule of law which permits the employer to be sued in England;^47^ and (viii) if the employer enters an appearance without contesting jurisdiction.^48^

On the other hand, there is another set of jurisdiction rules applicable when employees act as *defendants*. The employee may be sued by the employer in England: (i) if the employee is domiciled in England; *^49^ (ii) on a counterclaim, if the original claim against the employer is pending in England under rules (i)–(iii) from the preceding paragraph;^50^ (iii) if the employee is domiciled outside the UK, under any other rule of law which permits the employee to be sued in England;^51^ and (iv) if the employee enters an appearance without contesting jurisdiction,^52^ provided they were informed by the court of their right to contest the jurisdiction and the consequences of entering or not entering an appearance.^53^

These rules are subject to the rule of non-consensual exclusive jurisdiction in rule 11 of schedule 4.^54^ The rules marked with an asterisk (*) may be departed from only by a jurisdiction agreement which is entered into after the dispute has arisen or allows the employee to bring proceedings in courts other than those indicated in these rules.^55^ If the defendant is domiciled in another part of the UK and does not enter an appearance, an English court must declare of its own motion that it has no jurisdiction, unless it has jurisdiction by virtue of these rules.^56^ In such circumstances, the court must also stay the proceedings so long as it is not shown that the defendant has been able to receive the document instituting the proceedings or an equivalent document in sufficient time to enable them to arrange for their defence or all necessary steps have been taken to this end.^57^ Provisional measures may be sought in England under English law even if the courts of another part of the UK have jurisdiction as to the substance of the matter.^58^ Courts should have regard to the Court of Justice of the EU (CJEU)’s pre-Brexit case law and may consider the expert reports relating to the Brussels Convention when determining any question as to the meaning and effect of these rules.^59^

Rules on service in and outside the jurisdiction have also been amended to the effect that permission to serve out is not required where each claim made against the defendant to be served and included in the claim form is a claim which the court has power to determine under the CJJA 1982 rules, where no proceedings between the parties concerning the same claim are pending in the courts of any other part of the UK and where the defendant is an employer and a party to a contract of employment.^60^

Rule 10 of the Employment Tribunal Procedure Rules 2024^61^ (ETPR 2024) has succeeded rule 8 ETRP 2013. The two rules are essentially identical. Rule 10 ETPR 2024 is headed ‘Presenting the claim’ and is contained in the second part of the procedure rules, headed ‘Starting a claim’. Rule 10(2) provides that a claim may be presented in England if: (i) the respondent resides in England;^62^ (ii) the respondent carries on business in England;^63^(iii) one of the respondents resides in England;^64^ (iv) one of the respondents carries on business in England;^65^ (v) an act or omission complained of took place in England;^66^ (vi) the work under the contract to which the claim relates is or has been performed partly in England;^67^ and (vii) the case falls within the territorial scope of a statutory employment right and there is a connection with England.^68^ There are essentially identical provisions regulating the jurisdiction of employment tribunals in Scotland in rule 10(3),^69^ as well as of industrial tribunals in Northern Ireland in a separate instrument.^70^ Rule 98 ETPR 2024 provides for the transfer of proceedings between England and Scotland.^71^

At the same time, the Employment Appeal Tribunal has recently stated on two occasions that the fulfilment of the territorial scope requirement confers international jurisdiction, unless qualified or displaced by primary legislation.^72^

The co-existence of the CJJA 1982 rules with rules on service in and outside the jurisdiction and rules determining the jurisdiction of employment tribunals raises a number of questions about the relationship between the constituent elements of the current law.

## 3. Do CJJA 1982 Rules Apply before Employment Tribunals?

This question raises three sub-questions. Do the CJJA 1982 rules apply before employment tribunals in general? Do they apply to all types of claims that can be brought before employment tribunals? Do all CJJA 1982 rules apply before employment tribunals?

### A. In General?

Employment tribunals regularly hear cases with a foreign element. It may, therefore, appear surprising that one may ask, five years after the entry into force of the CJJA 1982 rules, whether they even apply before employment tribunals. Yet it is necessary to ask this question because the leading academic accounts of the English private international law of employment express diametrically opposite views.

Professor Merrett, the author of a leading monograph^73^ and several influential articles in the field,^74^ takes the view that the CJJA 1982 rules only apply to determine the jurisdiction of the civil courts and employment tribunals under their extended cause-of-action jurisdiction to hear certain contract claims.^75^ Professors Collins, Ewing and McColgan, the authors of a leading textbook on labour law, express a similar view.^76^

On the other hand, for the editors of *Dicey, Morris and Collins* and Professor Briggs, the application of the CJJA 1982 rules across the board, that is, before both the civil courts and employment tribunals regardless of the nature of the claim, seems to be so obvious that they do not discuss the potential role of any other rules for determining the jurisdiction of employment tribunals.^77^

These views are irreconcilable. The answer lies in reading the CJJA 1982 rules in light of section 50 CJJA 1982, which contains definitions of various terms used in the Act. The CJJA 1982 rules regulate the jurisdiction of ‘courts’. According to section 50, ‘In this Act, unless the context otherwise requires … “court”, without more, includes a tribunal’, ‘“tribunal” … means a tribunal of any description other than a court of law’ and ‘court of law’ means the Supreme Court and ‘in England and Wales, the Court of Appeal, the High Court, the Crown Court, the family court, the county court and a magistrates’ court’. Furthermore, the goal of the CJJA 1982 rules is to transpose the Brussels Ia Regulation’s rules of jurisdiction in employment matters. Since the regulation applies ‘in civil and commercial matters whatever the nature of the court or tribunal’,^78^ this goal can be achieved only if the term ‘courts’ in the CJJA 1982 rules is interpreted to cover employment tribunals.

This view is supported by case law. In none of the four judgments of the Employment Appeal Tribunal on the question of jurisdiction delivered in cases brought after Brexit was there any indication that the CJJA 1982 rules were inapplicable on the basis that they do not apply before employment tribunals. In fact, these judgments suggest the opposite.^79^

### B. To All Types of Claims?

Employment tribunals hear statutory claims and certain contract claims under their extended cause-of-action jurisdiction. The vast majority of these claims fall within the subject-matter scope of the CJJA 1982 rules. However, some do not.

One example is offered by cases of discrimination by not offering employment, prohibited by section 39(1)(c) of the Equality Act 2010 (EqA 2010). The CJJA 1982 rules apply ‘in relation to proceedings whose subject-matter is a matter relating to an individual contract of employment’.^80^ In *Fonderie Officine Meccaniche Tacconi SpA v Heinrich Wagner Sinto Maschinenfabrik GmbH*,^81^ the CJEU held that an action for breach of an obligation to negotiate in good faith was not a matter relating to a contract within the meaning of what is now article 7(1) of the Brussels Ia Regulation. This judgment suggests that a claim for breach of the obligation not to discriminate by not offering employment does not fall within the subject-matter scope of the regulation’s rules of jurisdiction in employment matters^82^ and, consequently,^83^ the CJJA 1982 rules.^84^

Another example is offered by claims against third parties, such as the employer’s employees and agents and persons instructing, causing, inducing or knowingly aiding contraventions of the EqA 2010 under sections 110–12 of the Act and persons treated as employers for the purposes of the right not to be subject to detriment arising from making a protected disclosure under section 43 K(2) ERA 1996 who do not satisfy the definition of ‘employer’ for the purposes of the CJJA 1982 rules.^85^

### C. To All CJJA 1982 Rules?

The fact that the vast majority of claims that can be brought before employment tribunals falls within the subject-matter scope of the CJJA 1982 rules does not necessarily mean that all CJJA 1982 rules apply to such claims. Section 15C preserves the application of the rule of non-consensual exclusive jurisdiction in rule 11 of schedule 4,^86^ the rule of jurisdiction over counterclaims in rule 5(c) of schedule 4,^87^ the rule of jurisdiction over branches, agencies or other establishments in rule 3(e) of schedule 4^88^ and the rule of jurisdiction over co-defendants in rule 5(a) of schedule 4.^89^ However, according to section 16(1), the provisions of schedule 4 have effect for determining whether ‘the courts of law’ of different parts of the UK have jurisdiction. Since employment tribunals are not ‘courts of law’,^90^ the employment tribunal has held that these jurisdiction rules cannot be invoked where a statutory claim is brought in an employment tribunal.^91^

## 4. Do CJJA 1982 Rules Exclusively and Exhaustively Determine International Jurisdiction of Employment Tribunals?

If the CJJA 1982 rules apply before both the civil courts and employment tribunals, there are two possibilities: first, that they exclusively and exhaustively determine the international jurisdiction of employment tribunals; and second, that they are permissive and employees can invoke other rules to establish the international jurisdiction of employment tribunals.

The leading academic accounts of the English private international law of employment are unhelpful. As explained in section 3A above, the view of Professors Merrett, Collins, Ewing and McColgan that the CJJA 1982 rules do not apply to determine the jurisdiction of employment tribunals to hear statutory claims is questionable. The editors of *Dicey, Morris and Collins* and Professor Briggs do not discuss the potential role of any rules other than the CJJA 1982 rules for determining the jurisdiction of employment tribunals.

Some employment tribunals adopt the view that the CJJA 1982 rules are exclusive and exhaustive. For example, the Employment Appeal Tribunal stated in *Stena Drilling* that:

For cases involving individual contracts of employment commenced after 31 December 2020, [sections 15C and 15D CJJA 1982] represent the only route through which international jurisdiction may be established. They replace the grounds of international jurisdiction which formerly applied within the United Kingdom by virtue of the recast Brussels Regulation 1215/2012.^92^

However, this judgment cannot be treated as resolving the matter once and for all, since the respondent’s argument that international jurisdiction could only be established under the CJJA 1982 rules was conceded by the claimant.^93^

The CJJA 1982 rules can only be exclusive and exhaustive if at least one of the following two conditions is met: (i) the CJJA 1982 rules themselves provide that they exclusively and exhaustively determine the international jurisdiction of employment tribunals; or (ii) there are no other rules regulating the international jurisdiction of employment tribunals.

### A. Do CJJA 1982 Rules Themselves Provide They Are Exclusive and Exhaustive?

The goal of the CJJA 1982 rules is to transpose the Brussels Ia Regulation’s rules of jurisdiction in employment matters. A feature of the latter rules is that they constitute a complete and self-contained ‘code’ for cases in which they apply. In other words, where the defendant is domiciled in an EU Member State, it can be sued in the courts of Member States only under the regulation’s rules of jurisdiction in employment matters. Neither the regulation’s generally applicable jurisdiction rules not referred to by the special rules of jurisdiction in employment matters^94^ nor jurisdiction rules in the national laws of Member States are applicable.^95^ Furthermore, even where article 21(1)(b) of the regulation confers jurisdiction over a defendant not domiciled in the EU on the courts of the Member State where the work is habitually performed/engaging place of business is situated, jurisdiction rules in the national laws of Member States are inapplicable.^96^

However, this is not a feature that the CJJA 1982 rules have retained. Section 15C(5)(d) provides that subsections (2) and (3) do not affect the operation of any other rule of law which permits a person not domiciled in the UK to be sued in the courts of a part of the UK. Consequently, the view that the CJJA 1982 rules are exclusive and exhaustive can only be correct if there are no other rules regulating the international jurisdiction of employment tribunals.

### B. Are There Other Rules Regulating International Jurisdiction of Employment Tribunals?

To address this question, a brief history of rule 10 ETPR 2024 and its predecessors needs to be presented.

Employment tribunals have been in operation since 1964 (before 1998, they were known as industrial tribunals). Since that time, procedure before employment tribunals has been regulated by a series of statutory instruments. Until 2004, there were separate procedure rules for proceedings commenced in employment tribunals in different parts of the UK. Since 2004, procedure before employment tribunals in England and Scotland has been regulated by one set of procedure rules.

Rule 10 ETPR 2024 is essentially identical to its predecessor, rule 8 ETRP 2013. The main difference between the two is that the ETPR 2024 are contained in a statutory instrument that only sets out the procedure rules of employment tribunals, whereas the ETRP 2013 were annexed as schedule 1 to a statutory instrument setting out both the constitution and procedure rules of employment tribunals.

The predecessor of rule 8 ETRP 2013 was regulation 19 ET(CRP)R 2004, which was different from its successor in four ways. First, it was contained in the main body of the ET(CRP)R 2004. Second, it was headed ‘Jurisdiction of tribunals in Scotland and in England & Wales’. Third, it was worded differently. It provided that ‘An employment tribunal in England or Wales shall only have jurisdiction to deal with proceedings’ in circumstances mentioned in regulation 19.^97^ Fourth, some of the bases of jurisdiction in regulation 19 were different.^98^

The predecessors of regulation 19 ET(CRP)R 2004 in the five sets of preceding procedure rules were similar to regulation 19, with the main differences being that they did not expressly provide that they dealt with the question of jurisdiction and they only applied to English courts. They were all contained in the main body of the relevant statutory instruments, headed ‘Proceedings of tribunals’.^99^

The predecessors of rule 10 ETPR 2024 were applied to determine the international jurisdiction of employment tribunals. In *Tweddell v Irish Shipping Ltd*,^100^ the industrial tribunal held that it did not have jurisdiction under regulation 3 of the Industrial Tribunals (Labour Relations) Regulations 1974 because the Irish respondents did not reside or carry on business in England and the cause of action arose in Scotland. In *Odeco (UK) Inc v Peacham*,^101^ the Employment Appeal Tribunal held that a non-UK company, which had a registered office in England and an operational base in Scotland, could be sued in England under regulation 3. In *Knulty v Eloc Electro-Optiek and Communicatie BV*,^102^ the industrial tribunal held that it had jurisdiction over a claim for unfair dismissal and redundancy payment under regulation 3 because the Dutch respondents carried on business in England and because the dismissal took place in England.

In *Lawson*,^103^ the House of Lords hinted that regulation 11(5) of the Employment Tribunals (Constitution and Rules of Procedure) Rules 2001 (ET(CRP)R 2001) dealt with the question of international jurisdiction.

In *Ritchie v Shawcor Inc*, a claim brought against non-UK respondents, the Employment Appeal Tribunal stated that:

In addition to the requirements of the particular statute on which the claim is based, in this case TUPE [Transfer of Undertakings (Protection of Employment) Regulations 1981, SI 1981/1794], an employment tribunal only has jurisdiction to deal with proceedings if regulation 19 [ET(CRP)R 2004] is satisfied.^104^

The respondents conceded the regulation 19 point.^105^ In *Hasan v Shell International Shipping Services (PTE) Ltd*,^106^ the Employment Appeal Tribunal held that it did not have ‘international jurisdiction’^107^ under regulation 19 over a Singaporean respondent because it did not reside or carry on business in England and because breach of contract occurred outside the jurisdiction.

The most important decision from this period is the Employment Appeal Tribunal judgment in *Pervez*.^108^ The employee worked for a Hong Kong member of a multinational group. His home and working base were in Hong Kong and there was a Hong Kong choice-of-law clause. He was seconded to London to another member of the multinational group, with the Hong Kong group member remaining the employer. Following the termination of his employment, the employee commenced proceedings in England. The unique feature of this case was that it fell within the territorial scope of statutory employment rights, but it appeared that none of the bases of jurisdiction in regulation 19 ET(CRP)R 2004 could be invoked against the foreign respondent. Underhill P noted that the purpose of regulation 19 was not simply to regulate intra-UK jurisdiction. The wording of regulation 19(1)(b), whose

broad effect is that the employment tribunal will have jurisdiction in respect of a particular claim if the acts or omissions which it is necessary to establish in order to constitute a cause of action, or any part of them, are alleged to have occurred in England or Wales

suggested that it regulated the jurisdiction of employment tribunals for cases with a ‘“non-GB” element’.^109^ The judge accepted that the foreign respondent

could not in any ordinary sense of the phrase be said to have been carrying on business in London. But if it followed that the tribunal had no jurisdiction that would be a very surprising result. Parliament would have conferred rights on a group of employees but would in respect of one sub-set of that group have failed to provide a forum in which those rights could be enforced.^110^

Consequently, Underhill P adopted a ‘strained construction’ of regulation 19 under which a foreign company could carry on business in England by seconding an employee to work at an establishment there.^111^

Following *Pervez*, Underhill J, then President of the Employment Appeal Tribunal, undertook the review of employment tribunal procedure rules in 2012/13 and devised the ETRP 2013.^112^ He tried to avoid the need for a ‘strained construction’ of jurisdiction rules in employment tribunal procedure rules by introducing rule 8(2)(d) ETRP 2013, which provided that a claim might be presented in England if ‘the Tribunal has jurisdiction to determine the claim by virtue of a connection with Great Britain and the connection in question is at least partly a connection with England and Wales’. As Brown J explains in *Zishaan v NCR Corp*:

Underhill J made clear in *Pervez*, in relation to r.19 of the ET Rules 2004 … that ... the wording of the rule had the effect of conferring international jurisdiction on the employment tribunal for cases with a ‘non-GB’ element …

*R.8 ET Rules 2013*, which Underhill J was responsible for drafting, even more clearly confers jurisdiction for claims with a foreign element, because only part of the claim requires a connection with GB*, r.8(2)(a) to (d).*

Therefore, in interpreting the 2013 rules, it is relevant that there is an express contemplation of a potential foreign element in ET proceedings and, specifically, proceedings against persons outside the UK in *rule 8*.^113^

There are cases brought before Brexit where employment tribunals stated that rule 8 ETRP 2013 determined their international jurisdiction where the jurisdiction rules of the Brussels Ia Regulation and the 2007 Lugano Convention did not apply.^114^

Therefore, the history of rule 10 ETPR 2024 and its predecessors shows a line, from 1974 until the adoption of the CJJA 1982 rules, of jurisdiction rules in employment tribunal procedure rules being used as international jurisdiction rules. This is a strong argument that there are rules other than the CJJA 1982 rules regulating the international jurisdiction of employment tribunals and that these other rules are contained in rule 10 ETPR 2024.^115^

Nevertheless, the recent judgments of the Employment Appeal Tribunal in *Bhatti*^116^ and *Lapinski*^117^ express the view that the fulfilment of the territorial scope requirement is capable of conferring international jurisdiction and that the identically worded predecessor of rule 10 ETPR 2024, rule 8 ETRP 2013, did not regulate the international jurisdiction of employment tribunals.^118^

In *Bhatti*, Kerr J accepted that

domestic statutes which have territorial application to a claim do themselves confer international jurisdiction, but only if that jurisdiction is not displaced by the Brussels Regulation or some other principle of private international law with force equal to that of primary domestic legislation, such as the relevant provisions of the 1982 Act.^119^

Furthermore, he did not

accept that a rule of procedure can itself confer jurisdiction where otherwise there is none. Rule 8 [ETRP 2013] is merely a procedural rule directing a claimant whether to present the claim in England and Wales (rule 8(2)) or in Scotland (rule 8(3)). In international jurisdiction cases, that depends on whether the sufficient connection is with England and Wales, or with Scotland.

… the tribunal must have, independently of rule 8(2)(d) (or 8(3)(d) for Scotland) international jurisdiction to determine it.^120^

In *Lapinski*, Auerbach J agreed with Kerr J.^121^ He supported the conclusion regarding rule 8 ETRP 2013 with additional arguments. First, in *Jackson v Ghost Ltd*^122^ and *Financial Times Ltd v Bishop*,^123^ the Employment Appeal Tribunal took the view that regulation 11(5) ET(CRP)R 2001, the twice-removed predecessor of rule 8 ETRP 2013, ‘was concerned merely with whether a given claim, in respect of which territorial jurisdiction existed, should be brought in England & Wales or in Scotland’.^124^ Second, ‘Rule 8 [ETRP 2013] forms part of the Rules of Procedure, not the governing regulations’.^125^ Third, ‘Within those Rules, it forms part of a section headed “Starting a Claim” and is headed “Presenting the claim”’.^126^ Fourth, ‘While rule 8(1) stipulates that a claim “shall” be started by presenting a claim as indicated there, rules 8(2) and (3) each indicate that it “may” be presented as provided there’.^127^ Fifth, the predecessors of rule 8(2) and (3) ETRP 2013, regulation 19(1) and (2) ET(CRP)R 2004, were worded differently. They ‘stipulated that the tribunal “shall only have jurisdiction” to deal with proceedings in England & Wales (or Scotland) if the following provisions applied’.^128^

By contrast rule 8 contains no wording to the effect that the tribunal ‘shall only have jurisdiction’. Rather, rules 8(2)(d) and 8(3)(d), in terms, do not determine jurisdiction, but apply in a case where the tribunal *does* have territorial jurisdiction by virtue of a connection with Great Britain.^129^

This led Auerbach J to conclude ‘that, while rule 8(2)(d) and 8(3)(d) do not confer jurisdiction, if it does not otherwise exist, nor do they [sic] rob the tribunal of jurisdiction, if it otherwise does’.^130^

One possible additional argument against the interpretation of rule 10 ETPR 2024 as regulating the international jurisdiction of employment tribunals is that schedule A1 to the ETA 1996, which makes further provision about employment tribunal procedure rules, does not expressly empower the Tribunal Procedure Committee to regulate international jurisdiction.^131^

However, the analyses in *Bhatti* and *Lapinski* are unpersuasive for the following reasons. First, as explained in section 2C above, the ‘principle’ *ubi jus, ibi remedium* rests on shaky foundations. Second, Kerr J’s analysis of this ‘principle’ and rule 8 ETRP 2013 was *obiter*, since the Brussels Ia Regulation applied and conferred jurisdiction on English courts in *Bhatti*.^132^ Third, the statement that the fulfilment of the territorial scope requirement confers international jurisdiction unless displaced, *inter alia*, by the Brussels Ia Regulation is legally incorrect. The regulation’s rules of jurisdiction in employment matters constitute a complete and self-contained ‘code’ for cases in which they apply. In other words, jurisdiction rules in the national laws of EU Member States are inapplicable in such cases.^133^ Fourth, there is nothing unusual about procedural rules regulating international jurisdiction. For example, the Civil Procedure Rules (and before them the Rules of the Supreme Court), although manifestly procedural rules, have long been recognised as regulating international jurisdiction in the cases to which they refer. Fifth, as explained in *Pervez*, the Employment Appeal Tribunal in *Jackson* and *Bishop* did not have in mind the potential impact of the wording of regulation 11(5) ET(CRP)R 2001 on cases with a ‘non-GB’ element.^134^ Sixth, the argument that regulation 11(5) ‘was concerned merely with whether a given claim, in respect of which territorial jurisdiction existed, should be brought in England & Wales or in Scotland’ is factually incorrect, as procedure before employment tribunals in England and Scotland has been regulated by one set of procedure rules since 2004. Seventh, arguments relying on the literal and systemic interpretation of rule 10 ETPR 2024 and its predecessors do not explain the many cases, from 1974 until the adoption of the CJJA 1982 rules, where employment tribunals used jurisdiction rules in employment tribunal procedure rules, worded similarly to rule 10 ETPR 2024 and rule 8 ETRP 2013, as international jurisdiction rules. Eighth, rule 17 in schedule A1 to the ETA 1996 provides that employment tribunal procedure rules may confer on the tribunal such ancillary powers as are necessary for the proper discharge of its functions. This is broad enough to include powers to hear cases with a foreign element.

### C. Conclusions

The conclusions of section 4 are that the CJJA 1982 rules themselves do not provide that they exclusively and exhaustively determine the international jurisdiction of employment tribunals, that there are other rules regulating the international jurisdiction of employment tribunals and that these other rules are contained in rule 10 ETPR 2024.

This matters in cases where the CJJA 1982 rules do not give international jurisdiction to employment tribunals but rule 10 ETPR 2024 does. This is possible because the CJJA 1982 rules and rule 10 ETPR 2024 use different connecting factors. An example is offered by the facts of *Powell v OMV Exploration and Production Ltd*.^135^ The employee, who lived in England, was employed by a foreign company to work in Dubai three weeks out of four, with the fourth week on leave in England, when he did some supervisory work. Since the employer’s domicile and habitual place of work were outside the UK, section 15C CJJA 1982 would not have pointed to the UK, but rule 10(2)(c) and (2)(d) ETPR 2024 would have.

## 5. Which Rules Regulate Intra-UK Jurisdiction?

This section addresses the question of whether the CJJA 1982 rules preclude the application of rule 10 ETPR 2024 as a rule allocating jurisdiction to hear employment cases within the UK by regulating not only international jurisdiction, but also intra-UK jurisdiction.

To answer this question, it is necessary to analyse separately three different types of cases: first, international cases without intra-UK elements, that is, cases connected with England and a country outside the UK; second, intra-UK cases without international elements, that is, cases that, from the perspective of English courts, have a connection with Scotland and/or Northern Ireland; and third, mixed cases. With this in mind, the question from the beginning of this paragraph branches out into four questions. Do the CJJA 1982 rules regulate both international and intra-UK jurisdiction in international cases? Do they regulate intra-UK jurisdiction in intra-UK cases? Do they regulate both international and intra-UK jurisdiction in mixed cases? If the answer to any of these questions is positive, what is the relationship between the CJJA 1982 rules and rule 10 ETPR 2024?

### A. International Cases

There are cases where employment tribunals have applied first the CJJA 1982 rules to determine whether they have international jurisdiction and then jurisdiction rules in employment tribunal procedure rules to decide whether they are the right venue within the UK in which to bring proceedings. For example, in *Bødker v MouldCAM Ltd*^136^ and *Singh v Singular Intelligence Ltd*,^137^ the employment tribunals held that section 15C(2)(a) CJJA 1982 gave jurisdiction to the courts of the employer’s domicile and that the employers were domiciled in the UK, after which they applied rule 8(2) ETRP 2013 and rule 10(2) ETPR 2024, respectively, to decide that they had jurisdiction.

This view is echoed in *Harvey on Industrial Relations and Employment Law*, whose editors claim that the CJJA 1982 rules only deal with international jurisdiction and that rule 10 ETPR 2024 only deals with intra-UK jurisdiction.^138^

However, the CJJA 1982 rules determine not only international jurisdiction but also intra-UK jurisdiction. Section 15C(2)(a)–(c) gives jurisdiction to the courts ‘for the part of the United Kingdom in which the employer is domiciled’ and the courts for the habitual place of work/engaging place of business in the UK, respectively.

### B. Intra-UK Cases

Section 16 CJJA 1982 provides that schedule 4, which contains a modified version of chapter 2 of the Brussels Ia Regulation and allocates jurisdiction in certain civil proceedings within the UK, does not apply for the purposes of determining jurisdiction in proceedings in relation to which section 15C or 15D(2) applies, except as specified in these sections.^139^ Since there is nothing in the CJJA 1982 rules to suggest that their application is excluded in intra-UK cases, it follows that the CJJA 1982 rules apply in such cases.^140^ In effect, the CJJA 1982 rules have replaced rule 10 of schedule 4,^141^ which dealt with ‘jurisdiction over individual contracts of employment’.

### C. Mixed Cases

If the CJJA 1982 rules regulate both international and intra-UK jurisdiction in international cases and if they allocate jurisdiction to hear employment cases within the UK in intra-UK cases, they must also regulate both international and intra-UK jurisdiction in mixed cases.

### D. Relationship between CJJA 1982 Rules and Rule 10 ETPR 2024

Where the jurisdiction of an employment tribunal is founded on a basis of jurisdiction set out or referred to in section 15C or 15D CJJA 1982 rules, there is no space for the application of rule 10 ETPR 2024. The only exception is section 15C(5)(d), which provides that subsections (2) and (3) do not affect the operation of any other rule of law which permits a non-UK-domiciled defendant to be sued in the courts of a part of the UK. In other words, in international and mixed cases, rule 10 ETPR 2024 can be applied as a rule allocating jurisdiction to hear employment cases within the UK only in cases falling outside the subject-matter scope of the CJJA 1982 rules, such as cases of discrimination by not offering employment and claims against third parties who do not satisfy the definition of ‘employer’ for the purposes of the CJJA 1982 rules, or where rule 10 ETPR 2024 is invoked under section 15C(5)(d) CJJA 1982 rules.

Moreover, section 15D(6)(a), which applies where a defendant domiciled in one part of the UK is sued in another part of the UK and does not enter an appearance,^142^ provides that the court must declare of its own motion that it has no jurisdiction, unless it has jurisdiction by virtue of section 15C or a rule from schedule 4 referred to in section 15C(4) or (5). This indicates that, in intra-UK cases and mixed cases where the defendant is domiciled in the UK, there is no space for the application of rule 10 ETPR 2024. In other words, in such cases, rule 10 can be applied as a rule allocating jurisdiction to hear employment cases within the UK only in cases falling outside the subject-matter scope of the CJJA 1982 rules.

## 6. How Should Courts Deal with Defective Transposition of Brussels Ia Regulation’s Rules?

The Brussels Ia Regulation’s rules of jurisdiction in employment matters have not been faithfully transposed. This is for two reasons.

First, the rule of jurisdiction over co-defendants in article 8(1) of the Brussels Ia Regulation provides that a person domiciled in an EU Member State may also be sued where he is one of a number of defendants, in the courts for the place where any one of them is domiciled, provided the claims are sufficiently closely connected. This rule is not contained in section 5 of chapter 2 of the regulation, but is applicable in proceedings brought by an employee by virtue of an express reference in article 20(1).

Section 15C(5)(c) CJJA 1982 preserves the application of the rule of jurisdiction over co-defendants in rule 5(a) of schedule 4 so far as it permits an employer to be sued by an employee. This rule provides that a person domiciled in a part of the UK may, in another part of the UK, also be sued where he is one of a number of defendants, in the courts for the place where any one of them is domiciled, provided the claims are sufficiently closely connected.

Whilst article 8(1) of the Brussels Ia Regulation and rule 5(a) of schedule 4 CJJA 1982 look similar, they are very different provisions. Article 8(1) of the Brussels Ia Regulation allocates jurisdiction among EU Member States. It allows the consolidation of disputes involving multiple defendants domiciled in two or more Member States before the courts of one of them. On the other hand, rule 5(a) of schedule 4 CJJA 1982 allocates jurisdiction in certain civil proceedings within the UK. It allows the consolidation of disputes involving multiple defendants before the courts of one part of the UK only where the anchor defendant and the defendant that the claimant wishes to join to the proceedings are both domiciled in the UK. In other words, this is a rule of intra-UK jurisdiction that cannot be invoked if either the anchor defendant or the defendant that the claimant wishes to join to the proceedings is not domiciled in the UK. This is problematic because the availability of a rule of international jurisdiction over co-defendants is important where an employee wishes to sue multiple defendants, some of which are domiciled in the UK and some outside the UK.

Second, as explained in section 3C above, section 15C CJJA 1982 preserves the application of the rule of non-consensual exclusive jurisdiction in rule 11 of schedule 4, the rule of jurisdiction over counterclaims in rule 5(c) of schedule 4, the rule of jurisdiction over branches, agencies or other establishments in rule 3(e) of schedule 4 and the rule of jurisdiction over co-defendants in rule 5(a) of schedule 4. However, these jurisdiction rules cannot be invoked where a statutory claim is brought in an employment tribunal. The inapplicability of the rule of jurisdiction over co-defendants in rule 5(a) of schedule 4 where a statutory claim is brought in an employment tribunal is problematic because the availability of a rule of jurisdiction over co-defendants is important where an employee wishes to sue multiple defendants domiciled in different parts of the UK. On the other hand, the inapplicability of the other jurisdiction rules listed above is not problematic because proceedings before employment tribunals do not concern non-consensual exclusive jurisdiction, employers cannot bring statutory claims and the definition of domicile has been extended under section 15C(7).

These problems are illustrated by *Lapinski*.^143^ The claimant was a member of a limited liability partnership. He commenced proceedings against the partnership and several individual respondents under the EqA 2010, alleging discrimination on the basis of a disability. Some respondents were domiciled and present in Sweden. This raised the question of whether the tribunal had jurisdiction over these respondents. The Employment Appeal Tribunal held that, although the claimant did not have a contract of employment with the partnership in the sense of domestic English law, this was an employment claim for the purposes of the CJJA 1982.^144^ However, the CJJA 1982 rules neither contain a rule of jurisdiction over non-UK-domiciled co-defendants nor refer to any such rule contained elsewhere. Rule 5(a) of schedule 4 was inapplicable, since this rule cannot be invoked where a statutory claim is brought in an employment tribunal and in any event the respondents that the claimant wished to join to the proceedings were not domiciled in the UK. If it was not possible to assume jurisdiction over the Swedish respondents, access to remedy and the enforcement of statutory employment rights would have been undermined.

That the CJJA 1982 rules neither contain a rule of jurisdiction over non-UK-domiciled co-defendants nor refer to any such rule contained elsewhere appears to be an oversight. Before Brexit, article 8(1) of the Brussels Ia Regulation applied in employment disputes. There is no indication in the Explanatory Memorandum^145^ that this oversight was deliberate. However, the goal of the CJJA 1982 rules to transpose the Brussels Ia Regulation’s rules of jurisdiction in employment matters cannot be invoked to confer jurisdiction under the Act where it otherwise does not exist, even where the omission of a jurisdiction rule is the result of an oversight. This conclusion is supported by the CJEU’s judgment in *Rouard.*^146^ Section 5 of chapter 2 of the Brussels I Regulation, the predecessor of the Brussels Ia Regulation, neither contained a rule of jurisdiction over co-defendants nor referred to the generally applicable rule of jurisdiction over co-defendants then found in article 6(1). In *Rouard*, the CJEU held that the generally applicable rule of jurisdiction over co-defendants could not be applied to a dispute falling within section 5 of chapter 2. To remedy this shortcoming, the Brussels Ia Regulation now includes an express reference to article 8(1) in article 20(1).

The defective transposition of the Brussels Ia Regulation did not prove to be an obstacle to the assumption of jurisdiction over the Swedish respondents in *Lapinski*.^147^ The Employment Appeal Tribunal applied the ‘principle’ *ubi jus, ibi remedium* and allowed the assumption of jurisdiction over the Swedish respondents because there was nothing in the CJJA 1982 rules to take away jurisdiction from the employment tribunal. As explained in section 2C above, the judgment in *Lapinski* may be criticised on the basis that this ‘principle’ rests on shaky foundations. Nevertheless, the outcome of this case is justified because, as section 4 concluded, there are rules other than the CJJA 1982 rules regulating the international jurisdiction of employment tribunals and these other rules are contained in rule 10 ETPR 2024, and they would have given jurisdiction over the Swedish respondents to the employment tribunal. Two provisions in rule 10 ETPR 2024 are particularly suitable for joining non-UK parties to the proceedings against a party over which the court has jurisdiction. First, rule 10(2)(a) provides that a claim may be presented in England if the respondent, or one of the respondents, resides or carries on business in England. The interpretation of the wording ‘or one of the respondents’ as enabling the consolidation of proceedings against multiple respondents where some do and some do not reside or carry on business in England is supported by the Employment Appeal Tribunal’s following observation in *Hasan*:

The reference to ‘the respondent or one of the respondents’ in the Regulation applies where there is an arguable claim against more than one respondent in respect of the same acts or omissions or in respect of joint and several liability where it is necessary and proper for joinder of more than one respondent.^148^

Second, rule 10(2)(d) provides that a claim may be presented in England if the case falls within the territorial scope of a statutory employment right and there is a connection with England.

By way of comparison, it should be noted that the jurisdiction of courts to hear common law claims depends on rules on service in and outside the jurisdiction. Claimants bringing such claims can invoke the gateway for necessary or proper parties in Civil Procedure Rules Practice Direction 6B, paragraph 3.1(3).

However, rule 10 ETPR 2024 and rules on service in and outside the jurisdiction cannot be applied to enable a party domiciled in another part of the UK to be joined to the proceedings against a non-UK-domiciled party over which the court has jurisdiction. This is because, by virtue of section 15D(6)(a) CJJA 1982, there is no space for the application of these rules where the defendant is domiciled in the UK and the rule of jurisdiction over co-defendants in rule 5(a) of schedule 4 is inapplicable where the anchor defendant is not domiciled in the UK.

## 7. *Can* Forum non Conveniens *Apply?*

There is uncertainty whether *forum non conveniens* can apply where the CJJA 1982 rules give jurisdiction to an English court and whether *forum non conveniens* can even be pleaded before employment tribunals. If *forum non conveniens* can apply, a separate question is whether the ability to make this plea makes any difference.

### A. *Can* Forum non Conveniens *Apply Where CJJA 1982 Rules Give Jurisdiction?*

Again, the reason why it is possible and necessary to ask this question is because the leading academic accounts of the English private international law of employment express diametrically opposite views.

Professors Merrett and Briggs are of the view that the doctrine is, as a matter of principle, inapplicable where the CJJA 1982 rules give jurisdiction to an English court.^149^ This view is supported by *obiter dicta* of the Employment Appeal Tribunal in *Lapinski*^150^ and the High Court made in the context of a discussion of the related rules of jurisdiction in consumer matters of the CJJA 1982 in *Alesayi v Bank Audi SAL*.^151^

On the other hand is the view of the editors of *Dicey, Morris and Collins*: ‘In the absence of any express provision to the contrary, it is tentatively suggested that the better view is that the application of the doctrine of *forum non conveniens* is, in principle, preserved.’^152^

A point that these accounts overlook is section 49 CJJA 1982. This section, headed ‘Saving for powers to stay, sist, strike out or dismiss proceedings’, provides:

Nothing in this Act shall prevent any court in the United Kingdom from staying, sisting, striking out or dismissing any proceedings before it, on the ground of *forum non conveniens* or otherwise, where to do so is not inconsistent with the 2005 Hague Convention.

Considering the clear statutory wording, it can only be concluded that the doctrine can apply where the CJJA 1982 rules give jurisdiction to an English court.

### B. *Can* Forum non Conveniens *Be Pleaded before Employment Tribunals?*

The doctrine can be pleaded before the civil courts. Since, within the confines of the 1994 Order, the jurisdiction of employment tribunals to hear certain contract claims is identical to, and concurrent with, that of the civil courts, the doctrine can also be pleaded where such a contract claim is brought before an employment tribunal.^153^ But can it be pleaded where a statutory claim is brought before an employment tribunal?

In *Lawson*, it was effectively common ground that an employment tribunal could stay its proceedings on the basis of *forum non conveniens*.^154^ This is unsurprising because rule 10(2)(h) of schedule 1 ET(CRP)R 2004 expressly provided that the chairman might, at any time, either on the application of a party or on his own initiative, make an order ‘staying (in Scotland, sisting) the whole or part of any proceedings’. There is no such provision in the ETPR 2024, or in the ETRP 2013 before it. Nevertheless, both the ETPR 2024 and the ETRP 2013 contain case management provisions, which provide that the tribunal may, on its own initiative or on the application of a party, make a case management order.^155^ They also provide that the particular powers identified in these rules do not restrict that general power.^156^ A case management order is defined as ‘an order or decision of any kind in relation to the conduct of proceedings, not including the determination of any issue which would be the subject of a judgment’.^157^ As Professor Merrett explains, ‘A stay of proceedings potentially comes within this broad definition and there is nothing to suggest that the changes were intended substantively to narrow the case management powers’.^158^ This appears to have been common ground before the Court of Appeal in *Asda Stores Ltd v Brierley* in relation to the ETRP 2013.^159^

### C. Does It Matter?

Even if the doctrine applies where the CJJA 1982 rules give jurisdiction to an English court and it can be pleaded before employment tribunals, the question arises whether the ability to make this plea makes any difference. For example, after stating that the doctrine is, in principle, preserved by the CJJA 1982 rules, the editors of *Dicey, Morris and Collins* write that ‘Nevertheless, it is difficult to envisage circumstances in which a foreign court would be regarded as more appropriate where the English court has jurisdiction under these provisions and so the point may be more of theoretical than practical significance’.^160^

It is indeed unlikely that the discretion to stay proceedings on the basis of *forum non conveniens* will ever be exercised by an employment tribunal in cases falling within the territorial scope of a statutory employment right, guaranteeing a real and substantial connection with the forum.^161^

Nevertheless, there are cases where the point is of practical significance. An example is offered by *Campbell v James Finlay (Kenya) Ltd*,^162^ where 5000 Kenyan tea pickers brought proceedings in Scotland against a Scottish company for occupational injuries in Kenya. Unusually for a transnational business and human rights dispute, the defender directly employed the claimants in Kenya. No Kenyan subsidiary or supplier was involved in the alleged wrongs. This enabled the pursuers to advance relatively straightforward negligence claims for breach of the employer’s duty of care. Everyone agreed that *prima facie* the court had jurisdiction under rule 1 in schedule 8 CJJA 1982, which is a Scottish rule of general jurisdiction based on the defender’s domicile. The defender challenged the court’s jurisdiction on, *inter alia*, *forum non conveniens* grounds. The Inner House of the Court of Session allowed the plea and sisted the proceedings. A glaring omission in the judgment is the court’s failure to acknowledge that in employment disputes the jurisdiction of UK courts depends on sections 15A and 15C–15E CJJA 1982.^163^ Had the court applied these rules, it would have found that Scottish courts had jurisdiction over the Scottish-domiciled defender.^164^ The key question would then have become the doctrine’s applicability.

## 8. What Are the Effects of Jurisdiction Agreements?

There are different rules regarding the effectiveness of jurisdiction agreements depending on a number of variables: whether the jurisdiction agreement prorogates or derogates the jurisdiction of English courts, the identity of the claimant, the nature of the claim, the judicial forum in which the claim is brought, the non-consensual basis of jurisdiction that would give jurisdiction to a UK court in the absence of an effective jurisdiction agreement and the defendant’s domicile.^165^

### A. Prorogation

Where an employee brings a claim falling within the subject-matter scope of the CJJA 1982 rules, an English jurisdiction agreement is effective, with one potential exception. If section 15C(2) gives jurisdiction to the courts of another part of the UK where the employer is domiciled or the habitual place of work/engaging place of business is situated, an English jurisdiction agreement is effective because section 15C(6)(b) allows the employee to bring proceedings in courts other than those indicated in section 15C(2). A civil court can (also) give effect to an English jurisdiction agreement against a non-UK employer under rule 6.33(2B) of the Civil Procedure Rules (CPR), which allows service outside the jurisdiction without the permission of the court where there is an English jurisdiction agreement and can be invoked by virtue of section 15C(5)(d) CJJA 1982. Since, within the confines of the 1994 Order, the jurisdiction of employment tribunals to hear certain contract claims is identical to, and concurrent with, that of the civil courts, an employment tribunal exercising its extended cause-of-action jurisdiction can (also) give effect to an English jurisdiction agreement against a non-UK employer following CPR 6.33(2B).

The potential exception to the effectiveness of English jurisdiction agreements where an employee brings a claim falling within the subject-matter scope of the CJJA 1982 rules is where a statutory claim is brought and section 15C(2) does not give jurisdiction to the courts of another part of the UK, that is, where the employer is domiciled outside the UK and the habitual place of work/engaging place of business is outside the UK. The reason why an English jurisdiction agreement is potentially ineffective in this scenario lies in the wording of section 15C(6), which appears to only deal with cases where section 15C(2) gives jurisdiction to the courts of a part of the UK and not lay down a more general rule regarding the effectiveness of jurisdiction agreements, and in the fact that CPR 6.33(2B) is unavailable because, with the exception of the exercise of extended cause-of-action jurisdiction, rules on service in and outside the jurisdiction are not relevant before employment tribunals. Nevertheless, this will usually not be problematic since rule 10(2)(d) ETPR 2024, which can be invoked by virtue of section 15C(5)(d) CJJA 1982, provides that a claim may be presented in England if the case falls within the territorial scope of a statutory employment right and there is a connection with England.

Where an employer brings a claim in a civil court falling within the subject-matter scope of the CJJA 1982 rules, the effectiveness of an English jurisdiction agreement is more limited. If section 15C(3) gives jurisdiction to the courts of another part of the UK where the employee is domiciled, an English jurisdiction agreement can only be effective under section 15C(6)(a), that is, if it is entered into after the dispute has arisen. A civil court can give effect to an English jurisdiction agreement against a non-UK employee following CPR 6.33(2B), which can be invoked by virtue of section 15C(5)(d) CJJA 1982.

If a common law claim falls outside the subject-matter scope of the CJJA 1982 rules, generally applicable private international law rules apply. If a statutory claim falls outside the subject-matter scope of the CJJA 1982 rules, such as a claim for breach of the obligation not to discriminate by not offering employment or a claim against a third party who does not satisfy the definition of ‘employer’ for the purposes of the CJJA 1982 rules, an English jurisdiction agreement is ineffective because there is nothing in rule 10 ETPR 2024 or elsewhere that provides for the prorogation of jurisdiction of employment tribunals. Nevertheless, this will usually not be problematic since rule 10(2)(d) provides that a claim may be presented in England if the case falls within the territorial scope of a statutory employment right and there is a connection with England.

### B. Derogation

The effectiveness of a foreign jurisdiction agreement depends on the non-consensual basis of jurisdiction that would give jurisdiction to English courts in the absence of an effective jurisdiction agreement.

If section 15C(2) gives jurisdiction to English courts where the employer is domiciled or the habitual place of work/engaging place of business is in England, the employer can invoke a foreign jurisdiction agreement only under section 15C(6)(a), that is, if it is entered into after the dispute has arisen. If section 15C(3) gives jurisdiction to English courts where the employee is domiciled in England, the employee can invoke a foreign jurisdiction agreement under section 15C(6)(b) because it allows the employee to bring proceedings in courts other than those indicated in this section.

If English courts have jurisdiction on another basis of jurisdiction, there is nothing in the CJJA 1982 to limit the effectiveness of a foreign jurisdiction agreement. In such cases, a foreign jurisdiction agreement is given effect as part of the *forum (non) conveniens* discretion. However, it is unlikely that this discretion will ever be exercised in cases falling within the territorial scope of a statutory employment right.^166^

## 9. Conclusions

The picture that emerges from the preceding discussion is one of considerable complexity, which is not fully reflected in either the existing accounts of the English private international law of employment or case law. Jurisdiction in employment matters depends on a number of variables, most importantly the identity of the claimant, the nature of the claim, the judicial forum in which the claim is brought, the defendant’s domicile and the existence of a jurisdiction agreement. This complexity is far from ideal. The lack of clarity on the relationship between the constituent elements of the current law of jurisdiction in employment matters has the potential to undermine access to remedy and the enforcement of employment rights in cases with a foreign element. Any revision of the current law would require consideration of four key issues.

First, what is the law of jurisdiction in employment matters for? Is it to protect UK-domiciled employees, employees connected with the UK in a broader range of ways or employees in general? The Explanatory Memorandum indicates that jurisdictional protection should neither be limited to UK-domiciled employees nor extended to employees in general.^167^

Given that the CJJA 1982 rules are derived from the Brussels Ia Regulation, jurisdictional protection should extend, at the very least, to UK-domiciled employees and those with a habitual place of work/engaging place of business in the UK. Whilst under the Brussels Ia Regulation there may be need for the habitual place of work to be a primary connecting factor and interpreted broadly and for the engaging place of business to be a secondary connecting factor and interpreted narrowly, the question arises whether employees engaged through a place of business in the UK should receive a broader jurisdictional protection than is currently provided by the CJJA 1982 rules. For example, if an English employee is engaged through a place of business there to habitually work for a non-UK-domiciled employer outside the UK, they cannot bring proceedings in the UK under the non-consensual bases of jurisdiction set out in the CJJA 1982 rules. It is true that an employee from one EU Member State who is engaged through a place of business there to habitually work for a foreign employer in another Member State cannot bring proceedings in the former Member State under the jurisdiction rule based on the connecting factor of the engaging place of business in the Brussels Ia Regulation. However, it is questionable whether this aspect of the regulation should have been transposed for two reasons. First, access to remedy and the enforcement of employment rights will not differ substantially from one Member State to another, given that much of employment law in the EU is derived from EU law and the European Convention on Human Rights, and that choice-of-law rules for individual employment contracts are uniform throughout the EU. Consequently, it is less important whether proceedings are commenced in the courts for the habitual place of work or in the courts for the engaging place of business within the EU. Second, if an employee from one Member State is engaged through a place of business there to habitually work for a non-EU-domiciled employer outside the EU, they can invoke jurisdiction rules in the national law of that Member State, which may allow the employee to bring proceedings there. The jurisdictional protection given to employees engaged through a place of business in the UK by the CJJA 1982 rules could be extended by allowing them to invoke the jurisdiction rule based on the connecting factor of the engaging place of business even if there is a habitual place of work elsewhere.

Since the key to employee protection lies in the enforcement of statutory employment rights, the law of jurisdiction in employment matters should also protect employees in cases falling within the territorial scope of statutory employment rights.

Second, should the law of jurisdiction in employment matters protect both defendant and claimant employees? The Explanatory Memorandum indicates that jurisdictional protection should extend to both.^168^ Two suggestions flow from this. First, some limitations that are currently built into the CJJA 1982 rules, namely the unavailability of the rule of jurisdiction over co-defendants against non-UK-domiciled employers and the potential ineffectiveness of English jurisdiction agreements where a statutory claim is brought and section 15C(2) does not give jurisdiction to the courts of another part of the UK, are not justified. The availability of the rule of jurisdiction over co-defendants against non-UK-domiciled employers would be particularly important as it would allow claimants in cases like *Lapinski*^169^ to consolidate disputes involving multiple defendants in England without having to rely on the ‘principle’ *ubi jus, ibi remedium*, which rests on shaky foundations. Second, all foreign jurisdiction agreements that potentially limit the jurisdictional options of claimant employees could be regulated by the CJJA 1982 and not the *forum (non) conveniens* discretion.

Third, should the law of jurisdiction in employment matters depend on the nature of the claim and the nature of the forum in which the claim is brought? There is no indication in the Explanatory Memorandum that this should be the case. Four suggestions flow from this. First, given that the lack of clarity is largely caused by the coexistence of the CJJA 1982 rules and rule 10 ETPR 2024, there is a strong argument for a simplification of the law. Since the recent judgments of the Employment Appeal Tribunal in *Bhatti*^170^ and *Lapinski* express the view that the identically worded predecessor of rule 10 ETPR 2024, rule 8 ETRP 2013, did not regulate the international jurisdiction of employment tribunals and since the CJJA 1982 rules regulate intra-UK jurisdiction, all rules of jurisdiction in employment matters could be contained in the CJJA 1982. Second, the subject-matter scope of the CJJA 1982 rules could be extended to cover cases currently falling within the territorial scope of a statutory employment right but outside the subject-matter scope of the CJJA 1982 rules, such as cases of discrimination by not offering employment and claims against third parties who do not satisfy the definition of ‘employer’ for the purposes of the CJJA 1982 rules. Third, some limitations that are currently built into the CJJA 1982 rules, namely the unavailability of the rule of jurisdiction over co-defendants where a statutory claim is brought in an employment tribunal, as well as the potential ineffectiveness of English jurisdiction agreements where a statutory claim is brought and section 15C(2) does not give jurisdiction to the courts of another part of the UK, are not justified. As mentioned in the preceding paragraph, the availability of the rule of jurisdiction over co-defendants against non-UK-domiciled employers would be particularly important. Fourth, a rule providing that a claim may be presented in a part of the UK if the case falls within the territorial scope of a statutory employment right and there is a connection with that part of the UK could be introduced in the CJJA 1982 and modelled on rule 10(2)(d) ETPR 2024. This rule would provide jurisdictional protection to employees in cases falling within the territorial scope of statutory employment rights where the CJJA 1982 rules do not otherwise give jurisdiction to an English court and would obviate the need to rely on the ‘principle’ *ubi jus, ibi remedium*.

Finally, should *forum non conveniens* apply where the CJJA 1982 rules give jurisdiction to an English court? According to the Explanatory Memorandum, the goal of the CJJA 1982 rules is to continue ‘a right [of employees] to be sued … only in the part of the UK in which they are domiciled (regardless of the domicile of the other party), and a right to sue the other party … in parts of the UK with relevant connections’.^171^ According to the Employment Appeal Tribunal in *Lapinski*,^172^ this entails the unavailability of *forum non conveniens* under the CJJA 1982 rules. Accordingly, section 49 could be amended by carving sections 15A–15E out of the operation of this provision. This would affect cases such as *Campbell*.^173^ However, if intra-UK jurisdiction is exclusively regulated by the CJJA 1982, it should be considered whether a rule on the transfer of proceedings between employment tribunals in different parts of the UK, like the one in rule 98 ETPR 2024 but also covering Northern Ireland, could be introduced in the Act.

## Footnotes

1 Regulation 1215/2012 [2012] OJ L351/1, ch 2, s 5, replacing Regulation 44/2001 [2001] OJ L12/1, ch 2, s 5, which, in turn, replaced the Brussels Convention [1998] OJ C 27/1 (consolidated version), arts 5(1) and 17(5).

2 [2009] OJ L147/1, title 2, s 5, replacing the 1988 Lugano Convention [1988] OJ L319/9, arts 5(1) and 17(5).

3 Civil Jurisdiction and Judgments (Amendment) (EU Exit) Regulations 2019, SI 2019/479.

4 UK Government, ‘Explanatory Memorandum to the Civil Jurisdiction and Judgments (Amendment) (EU Exit) Regulations 2019’ para 7.10 <www.legislation.gov.uk/ukdsi/2019/9780111176726/pdfs/ukdsiem_9780111176726_en.pdf>.

5 This article focuses on the law of England and Wales. For the sake of simplicity, ‘England’ and ‘English’ is used instead of ‘England and Wales’ and ‘English and Welsh’. The insights of this article are also relevant for Scottish and Northern Irish laws.

6 Adrian Briggs, ‘The Hidden Depths of the Law of Jurisdiction’ [2016] LMCLQ 236.

7 See eg Employment Rights Act 1996, s 111 for unfair dismissal and Equality Act 2010, s 120 for a contravention of pt 5 (work) of the Act.

8 SI 1994/1623, adopted pursuant to ETA 1996, s 3, sch 2, para 2.

9 1994 Order, art 4.

10 Louise Merrett, ‘The Extra-Territorial Reach of Employment Legislation’ (2010) 39 ILJ 355, 357–9.

11
*Prähl v Lapinski* [2025] EAT 77 [12] (appeal pending).

12 [2012] ICR 1343 (EAT) [8]–[9].

13
*Lapinski* (n 11) [10].

14
*Serco Ltd v Lawson* [2006] UKHL 3 [24].

15
*Cable News International Inc v Bhatti* [2025] EAT 63 [60].

16 ibid.

17 (n 11).

18 (n 15) [65].

19 [2011] ICR 266 (EAT) [21]. See *Bhatti* (n 15) [19] and [60].

20 (n 14) [1]. See *Bhatti* (n 15) [60]–[65].

21 (n 11) [27]–[32], [34] and [63].

22 SI 2004/1861.

23 (n 14) [15] (emphasis added).

24 Providing that the claimant may serve the claim form out of the jurisdiction with the permission of the court where a claim is made under an enactment which allows proceedings to be brought and those proceedings are not covered by any of the other jurisdictional ‘gateways’.

25 See Louise Merrett, *Employment Contracts in Private International Law* (OUP 2011) chs 4 and 5; Amy Rogers and Daniel Oudkerk, *International Employment Disputes* (Sweet & Maxwell 2019) chs 4 and 5.

26 Brussels Ia Regulation, arts 4(1), 5, 6(1), 21(1)(b), 21(2), 24 and 25.

27 2007 Lugano Convention, arts 2(1), 3, 4(1), 22 and 23.

28 Brussels I Regulation, ch 2, s 5.

29 2007 Lugano Convention, title 2, s 5.

30 Brussels I Regulation, art 1(1); 2007 Lugano Convention, art 1(1).

31 ETA 1996, s 3(2) and (4); *Lyons v Interserve Group Ltd* (ET, 18 January 2021) [77]. However, employment tribunals cannot order the payment of an amount exceeding £25,000: 1994 Order, art 10.

32 Merrett, *Employment Contracts* (n 25) para 5.01; Rogers and Oudkerk (n 25) paras 4-19–4-27.

33 Merrett, *Employment Contracts* (n 25) paras 5.15–5.18; Thomas Linden, ‘Employment Litigation Involving Employees Who Work Abroad and/or Foreign Employers’ (Matrix Annual Employment Seminar, 7 February 2017) para 26 <www.matrixlaw.co.uk/wp-content/uploads/2017/02/Employment-Seminar-2017-Paper.pdf>; Rogers and Oudkerk (n 25) paras 5-02, 5-03 and 5-05–5-08.

34 SI 2013/1237.

35 Andrew Scott, ‘The Territorial Scope of British Employment Legislation’ [2010] LMCLQ 640, 646–7.

36 Merrett, *Employment Contracts* (n 25) paras 5.29–5.32.

37 European Commission, ‘Communication from the Commission to the European Parliament and the Council: Assessment on the Application of the United Kingdom of Great Britain and Northern Ireland to Accede to the 2007 Lugano Convention’ COM (2021) 222 final (4 May 2021).

38 (n 4) para 7.19.

39 CJJA 1982, s 15A(2), referring to Brussels Ia Regulation, art 1. For subject-matter exclusions, see CJJA 1982, s 15A(3), sch 5.

40 CJJA 1982, s 15C(1). Similarly, s 15A(1)(b).

41 ibid s 15C(2)(a). Domicile is defined in ss 42(4A) and 42A. An employer not domiciled in the UK under these rules is deemed to be domiciled in the part of the UK where it has a branch, agency or other establishment and the dispute arose from the operation of that establishment: s 15C(7).

42 ibid s 15C(2)(b). This rule is expressed to apply regardless of the employer’s domicile.

43 ibid s 15C(2)(c). This rule is expressed to apply regardless of the employer’s domicile.

44 ibid s 15C(5)(a). This rule can apply regardless of the employer’s domicile. This is the effect of the use of the wording ‘under rule 5(c) of Schedule 4 *or otherwise*’ in s 15C(5)(a) (emphasis added).

45 ibid s 15C(5)(b), referring to sch 4, r 3(e), which is a rule of intra-UK jurisdiction. Although, in theory, claimant employers can also invoke this jurisdiction rule, this is practically impossible, since employees do not have branches, agencies or other establishments.

46 ibid s 15C(5)(c), referring to sch 4, r 5(a), which is a rule of intra-UK jurisdiction.

47 ibid s 15C(5)(d).

48 ibid s 15D(2) and (3)(a).

49 ibid s 15C(3). Domicile is defined in s 41. English courts protect this right of employees to be sued in the courts of their domicile by anti-suit injunctions: *Gagliardi v Evolution Capital Management LLC* [2023] EWHC 1608 (Comm).

50 ibid s 15C(5)(a). This rule can apply regardless of the employee’s domicile: n 44 above.

51 ibid s 15C(5)(d).

52 ibid s 15D(2) and (3)(a).

53 ibid s 15D(4).

54 ibid ss 15C(4) and 15D(3)(b).

55 ibid s 15C(6)(a). Agreements or provisions of a trust instrument conferring jurisdiction are subject to the same limitations: s 15D(1).

56 ibid s 15D(5) and (6)(a).

57 ibid s 15D(6)(b).

58 ibid s 15D(7).

59 ibid s 15E(2).

60 Civil Procedure Rules 6.33(2).

61 SI 2024/1155.

62 ibid r 10(2)(a).

63 ibid.

64 ibid.

65 ibid.

66 ibid r 10(2)(b).

67 ibid r 10(2)(c).

68 ibid r 10(2)(d).

69 ibid r 10(3).

70 Industrial Tribunals and Fair Employment Tribunal (Constitution and Rules of Procedure) Regulations (Northern Ireland) 2020 SR 2020/3, sch 1.

71 No such rule exists with respect to Northern Ireland.

72
*Bhatti* (n 15) [59]–[65] and [71], affirmed in *Lapinski* (n 11) [24]–[35], [49]–[58] and [63].

73 Louise Merrett, *Employment Contracts in Private International Law* (2nd edn, OUP 2022).

74 Merrett, ‘Extra-Territorial Reach’ (n 10); Louise Merrett, ‘International Employment Cases Post-Brexit: Choice of Law, Territorial Scope, Jurisdiction and Enforcement’ (2021) 50 ILJ 343.

75 Merrett, *Employment Contracts* 2nd edn (n 73) paras 5.02 and 5.41.

76 Hugh Collins, KD Ewing and Aileen McColgan, *Labour Law* (3rd edn, CUP 2025) 61.

77 Lawrence Collins and Jonathan Harris (gen eds), *Dicey, Morris and Collins on the Conflict of Laws* (16th edn, Sweet & Maxwell 2022) paras 11-246 *et seq*; Adrian Briggs, *Private International Law in English Courts* (2nd edn, OUP 2023) 183 *et seq*. See also David McClean, *The Conflict of Laws* (11th edn, Sweet & Maxwell 2025) paras 5-064–5-065.

78 Brussels Ia Regulation, art 1(1).

79
*Yacht Management Co Ltd v Gordon* [2024] EAT 33 (Scotland) [28] and [30]; *Stena Drilling Pte Ltd v Smith* [2024] EAT 57 (Scotland) [14]; *Bhatti* (n 15) [64]–[65]; *Lapinski* (n 11) [65]–[67].

80 CJJA 1982, s 15C(1).

81 Case C-334/00 ECLI:EU:C:2002:499.

82 Uglješa Grušić, *The European Private International Law of Employment* (CUP 2015) 93; Merrett, *Employment Contracts* 2nd edn (n 73) para 4.91.

83 CJJA 1982, s 15E(2)(a).

84
*Wisniewski v Express VPN at Kape Technologies PLC* (ET, 26 October 2023) [112].

85 See eg *Davidsen v IBM United Kingdom Ltd* (ET, 21 May 2025) [115]–[130] (the parent company of the first respondent and two of its employees were not ‘employers’ for the purposes of the CJJA 1982 rules). See also Paul Nicholls, *Employment and Commercial Disputes: The International Aspects* (Bloomsbury Professional 2021) paras 14.21–14.22 and 14.33.

86 CJJA 1982, s 15C(4).

87 ibid s 15C(5)(a).

88 ibid s 15C(5)(b).

89 ibid s 15C(5)(c). The application of this rule is preserved so far as it permits an employer to be sued by an employee.

90 ibid s 50.

91
*Atalian Servest Ltd v Bidvest Noonan (UK) Ltd* (ET, 3 January 2024) [116]–[118].

92 (n 79) [14]. Similarly, [32]. This view was followed in *Macaulay v Holmes* (ET, 16 October 2024) [21]; *Stasiak v Austen Maritime PTE Ltd* (ET (Scotland), 1 May 2025) [80]; *Davidsen* (n 85) [92].

93 (n 79) [29].

94 Case C-462/06 *Glaxosmithkline v Rouard* ECLI:EU:C:2008:299.

95 Case C-804/19 *BU v Markt24 GmbH* ECLI:EU:C:2021:134.

96 Case C-604/20 *ROI Land Investments Ltd v FD* ECLI:EU:C:2022:807.

97 ET(CRP)R 2004, reg 19(1).

98 The respondent or one of the respondents resides or carries on business in England (ibid reg 19(1)(a)); had the remedy been by way of action in the county court, the cause of action would have arisen wholly or partly in England (reg 19(1)(b)); the proceedings are to determine a question which has been referred to the tribunal by a court in England (reg 19(1)(c)); in the case of proceedings to which sch 3, 4 or 5 applies, the proceedings relate to matters arising in England (reg 19(1)(d)). Para (2) regulated the jurisdiction of employment tribunals in Scotland and is identical to para (1), but with ‘Scotland’ substituted for ‘England and Wales’.

99 Employment Tribunals (Constitution and Rules of Procedure) Regulations 2001, SI 2001/1171, reg 11(5); Industrial Tribunals (Constitution and Rules of Procedure) Regulations 1993, SI 1993/2687, reg 8(3); Industrial Tribunals (Rules of Procedure) Regulations 1985, SI 1985/16, reg 3(1); Industrial Tribunals (Rules of Procedure) Regulations 1980, SI 1980/884, reg 3(1); Industrial Tribunals (Labour Relations) Regulations 1974, SI 1974/1386, reg 3.

100 (IT, 3 April 1979).

101 [1979] ICR 823 (EAT).

102 [1979] ICR 827 (IT) [18]–[20].

103 (n 14) [15], extracted in text accompanying n 23 above.

104 (EAT (Scotland), 6 March 2008) [18].

105 ibid [21].

106 (EAT, 14 January 2014) [30] and [38]–[41].

107 The Employment Appeal Tribunal used the term ‘international jurisdiction’ at ibid [40]. Linden also expressed a view that ET(CRP)R 2004, reg 19 dealt with international jurisdiction: Thomas Linden, ‘Employment Protection for Employees Working Abroad’ (2006) 35 ILJ 186, 194.

108 (n 19).

109 ibid [15].

110 ibid [19].

111 ibid [21].

112 Department for Business Innovation and Skills, ‘Employment Tribunal Rules: Review by Mr Justice Underhill—Consultation’ (September 2012) <https://assets.publishing.service.gov.uk/government/uploads/system/uploads/attachment_data/file/32696/12-1039-employment-tribunal-rules-underhill-review.pdf>.

113 (ET, 31 July 2020) [49]–[51].

114
*Bumbalov v Harding Brothers (Guernsey) Ltd* (ET, 20 March 2017) [4]–[5]; *Dunne v Language Link London Ltd* (ET, 19 November 2020) [41]–[42]; *Lyons* (n 31) [47]–[50] and [68]; *Haughey v Prosafe Offshore Employment Company PTE Ltd* (ET (Scotland), 2 February 2021) [72]–[74]; *Hourigan v Interserve Group Ltd* (ET, 22 July 2021) [27]–[29]. See also *Twistdx Ltd v Armes* [2024] EAT 45 [44]–[47], stating it is arguable that ETRP 2013, r 8 regulated international jurisdiction.

115 In cases brought after Brexit, only jurisdiction rules in employment tribunal procedure rules were applied to determine the international jurisdiction of employment tribunals in *Joynat v The English School for Girls* (ET, 1 February 2022) [9]–[10]; *Horgan v Chevron Transport Corp Ltd* (ET, 22 April 2022) [18]-[19]; *Harahap v Observatory Southeast Asia Ltd* (ET, 26 July 2023) [4]–[5]; *Foster v Leetent Ltd* (ET, 10 April 2025) [9]-[10]; *Issa v Relief International Syria* (ET, 22 September 2025) [9]-[14]. In *Nishat v Dart* (ET, 15 October 2021) [91]–[103], only jurisdiction rules in employment tribunal procedure rules were applied to determine the international jurisdiction of the employment tribunal over contract (not statutory) claims.

116 (n 15).

117 (n 11).

118 This view was followed in *Nazarenko v Total Energies Gestion Internationale SA* (ET, 13 January 2026). See also Nicholls (n 85) ch 14.

119 (n 15) [65].

120 ibid [75]–[76].

121 (n 11) [24]–[35], [49] and [63].

122 [2003] IRLR 824 (EAT).

123 (EAT, 25 November 2003).

124 (n 11) [51].

125 ibid [55].

126 ibid.

127 ibid.

128 ibid [56].

129 ibid [57].

130 ibid [58]. The editors of *Harvey on Industrial Relations and Employment Law* rely on most of these arguments to support their view that ETPR 2024, r 10 does not regulate the international jurisdiction of employment tribunals: Ian Smith (gen ed), *Harvey on Industrial Relations and Employment Law: Division PIII Jurisdiction* (contrib ed Thomas Cordrey, LexisNexis up to date 1 May 2025) paras 251–253.01.

131 By analogy with the arguments made in Nicholls (n 85) paras 14.11–4.12; *Harvey on Industrial Relations and Employment Law* (n 130) para 252.

132 (n 15) [77]–[94].

133
*Markt24* (n 95).

134 (n 19) [15](1).

135 [2014] ICR 63 (EAT).

136 (ET, 18 October 2023) [44]–[45].

137 (ET, 4 February 2025) [2]–[4].

138 (n 130) paras 251–253.01.

139 CJJA 1982, s 16(1A). Similarly, s 16(4).

140 Explanatory Memorandum (n 4) para 2.6; Adrian Briggs, *Civil Jurisdiction and Judgments* (8th edn, Informa Law 2025) 10.01–10.03, 10.15 and 10.27–10.29; *Dicey, Morris and Collins* (n 77) paras 11-275, 11-296 and 11-299 fn 876.

141 Civil Jurisdiction and Judgments (Amendment) (EU Exit) Regulations 2019, reg 59(a).

142 CJJA 1982, s 15D(5)(a).

143 (n 11).

144 ibid [70]–[74].

145 (n 4).

146 (n 94).

147 See also *Nazarenko* (n 118).

148 (n 106) [31]. cf *Smirnov v Network Rail Ltd* (ET, 9 March 2022) [19](a).

149 Merrett, *Employment Contracts* 2nd edn (n 73) para 5.70; Briggs, *Private International Law* (n 77) 194.

150 (n 11) [76]–[77].

151 [2025] EWHC 1033 (KB) [6].

152 (n 77) para 12-012.

153
*Zishaan* (n 113) [37].

154 (n 14) [24]; Linden, ‘Employment Protection (n 107) 195.

155 ETPR 2024, r 30(1). Similarly, ET(CRP)R 2013, r 29.

156 ETPR 2024, r 30(2). Similarly, ET(CRP)R 2013, r 29.

157 ETPR 2024, r 2(1). Similarly, ET(CRP)R 2013, r 1(3)(a).

158 Merrett, *Employment Contracts* 2nd edn (n 73) para 5.74.

159 [2016] EWCA Civ 566 [15].

160 (n 77) para 12-012 (footnote omitted). Similarly, Merrett, *Employment Contracts* 2nd edn (n 73) paras 5.68 and 5.71; Briggs, *Private International Law* (n 77) 194.

161
*Lawson* (n 14) [24].

162 [2023] CSIH 39.

163 Michiel Poesen, ‘A Scots Perspective on *Forum non Conveniens* in Business and Human Rights Litigation: *Hugh Campbell KC v James Finlay (Kenya) Ltd*’ (2025) 21 J Priv Int L 135.

164 CJJA 1982, s 15C(2)(a).

165 The Hague Convention of 30 June 2005 on Choice of Court Agreements does not apply to contracts of employment: art 2(1)(b).

166 See *Spence v Environmental Products and Services Ltd* (ET, 14 March 2024) [40].

167 (n 4) para 2.6.

168 ibid paras 2.6 and 7.1.

169 (n 11).

170 (n 15).

171 (n 4) para 2.6. See Case C-281/02 *Owusu v NB Jackson, t/a ‘Villa Holidays Bal-Inn Villas’* ECLI:EU:C:2005:120.

172 (n 11) [76]–[77].

173 (n 162).

